# Expressions of pandemic fatigue on digital platforms: a thematic analysis of sentiment and narratives for infodemic insights

**DOI:** 10.1186/s12889-024-17718-4

**Published:** 2024-03-05

**Authors:** Becky K. White, Atsuyoshi Ishizumi, Lucy Lavery, Amy Wright, Tom Foley, Rhys O’Neill, Kimberly Rambaud, Ravi Shankar Sreenath, Cristiana Salvi, Ryoko Takahashi, Marcelo D’Agostino, Tim Nguyen, Sylvie Briand, Tina D. Purnat

**Affiliations:** 1 Department of Pandemic and Epidemic Preparedness and Prevention, Health Emergencies Programme, World Health Organization, Geneva, Switzerland; 2 Marble Global LTD, London, UK; 3 Africa Infodemic Response Alliance, WHO Regional Office for Africa, Brazzaville, Democratic Republic of the Congo; 4 Risk Communication & Community Engagement, Health Emergencies, WHO Regional Office for Europe, Copenhagen, Denmark; 5 Information Systems for Health, Evidence and Intelligence for Action in Health, Pan American Health Organization and World Health Organization Regional Office for the Americas, Washington DC, DC USA

**Keywords:** Pandemic fatigue; COVID-19, Mpox, Infodemic, Preparedness, Prevention

## Abstract

**Background:**

The infodemic accompanying the COVID-19 pandemic has led to an overwhelming amount of information, including questions, concerns and misinformation. Pandemic fatigue has been identified as a concern from early in the pandemic. With new and ongoing health emergencies in 2022, it is important to understand how pandemic fatigue is being discussed and expressed by users on digital channels. This study aims to explore and report on key narrative themes associated with expressions of pandemic fatigue by users on digital platforms.

**Methods:**

This paper describes the collection of publicly available data over a 3-month period from multiple online sources using the Meltwater and CrowdTangle platforms to source data from Twitter, Facebook, Instagram, YouTube, TikTok, Pinterest, Product Reviews, Twitch, blogs & forums. A comprehensive search strategy was developed and tested. A total of 1,484,042 social media posts were identified during the time-period that included the defined search terms for pandemic fatigue. These data were initially sorted by highest levels of engagement and from this dataset, analysts reviewed the identified posts to isolate and remove irrelevant content and identify dominant narratives. A thematic analysis was carried out on these narratives to identify themes related to expression of pandemic fatigue. Two researchers reviewed the data and themes.

**Results:**

The thematic analysis of narratives identified six main themes relating to expression of pandemic fatigue, and one theme of counter narratives against pandemic fatigue. Data volume increased concurrent with the time of the mpox emergency announcement. Emergent themes showed the different ways users expressed pandemic fatigue and how it was interlaced with issues of trust, preventative measure acceptance and uptake, misinformation, and being overwhelmed with multiple or sustained emergencies.

**Conclusions:**

This paper has identified the different ways users express pandemic fatigue on digital channels over a 3-month period. Better understanding the implications of the information environment on user’s perceptions, questions, and concerns regarding pandemic and more broadly emergency fatigue is vital in identifying relevant interventions and, in the longer term, strengthening the global architecture for health emergency preparedness, prevention, readiness and resilience, as evidenced in this paper. There are clear pathways for further research, including incorporating additional languages and reviewing these themes over longer time periods.

**Supplementary Information:**

The online version contains supplementary material available at 10.1186/s12889-024-17718-4.

## Background

Since the start of 2020, the world has been experiencing the COVID-19 pandemic [1]. The implications of COVID-19 have been felt globally. Multiple public health and social measures (PHSM) have been introduced at different times, restrictions have been placed on movement and travel, and the development of COVID-19 vaccines has been observed in real time. As we have learnt more about the pandemic and what we can do to protect populations, people have been faced with an infodemic, an overwhelming amount of information, including misinformation, disinformation and rumours [2]. The infodemic can make it difficult for people to identify credible health information, lead to questions or concerns, and negatively affect their health behaviors, including with potentially fatal outcomes [3]. During the COVID-19 pandemic the sheer amount of information being shared on social and other media, as well as via offline community sources (such as community feedback data, or information from hotlines), has impacted people’s ability to be able to filter reliable and important information and to protect themselves in turn [4]. As a result, the infodemic may prolong or reduce effectiveness of the public health response to emergencies. As we look to future preparedness and prevention, evidence-based solutions to tackle the infodemic are needed [5].

### Pandemic fatigue

Pandemic fatigue has been defined in different ways in the literature. A 2020 WHO European Regional Office report defined pandemic fatigue as “demotivation to engage in protection behaviours and seek COVID-19-related information, and as complacency, alienation and hopelessness” [6]. Other papers have defined pandemic fatigue as the ‘notion of behavioural fatigue associated with adherence to covid restrictions’ [7] or as a type of behavioural fatigue ‘characterized by reduced compliance with public health directives over time’ [8]. How pandemic fatigue has been measured in the literature points to further different ways of defining. One study used comparisons of the use of protective measures at different time points as a proxy for pandemic fatigue [9]. It reported that younger people and being of male gender was predictive of pandemic fatigue (reduced protective measure use) highlighting this as a key focus for risk communication messaging [9]. Another study in the Central Philippines investigated the link between pandemic fatigue and front-line healthcare workers in terms of impact on resilience, mental health, and job contentment [10]. This study used a 10-item Lockdown / Pandemic Fatigue scale, covering feelings of worry, distress, loss of interest in usual activities and experience of headache, among others [11]. A cross-sectional study in Israel did not report a specific measure for pandemic fatigue, rather used a multivariate analysis to look at the impact of a range of factors on vaccine hesitancy [12]. The perception of the importance and efficacy of COVID-19 vaccines was a significant predictor of vaccine uptake, with the authors concluding this ‘effectively demonstrated pandemic fatigue is at play’ [12]. Evidence is emerging of the impact and prevalence of COVID-19-related pandemic fatigue. At the end of 2021 a Monmouth University Poll reported 60% of Americans felt ‘worn out’ by COVID-19, with 45% reporting feeling angry about how COVID-19 had impacted their lives [13].

### Global situation

Pandemic fatigue has been identified as a concern from very early on in the COVID-19 pandemic due to the potential impact on risk perceptions and adherence to recommended PHSM. In 2020, at the request of Member States the WHO Regional Office for Europe released a policy document on managing and mitigating pandemic fatigue [6]. The situation now, 3 years since the COVID-19 onset, is significantly more complex. COVID-19 continues to be a public health emergency with some countries experiencing higher case numbers and deaths in 2022 than at any other time [14]. New variants and frequency of COVID-19 vaccination recommendations has led to continued vaccine misinformation and vaccine hesitancy [15] and has affected routine immunization programmes [16]. There are reports of exhaustion in the healthcare workforce [17]. In 2022, mpox was declared a public health emergency of international concern (PHEIC) [18], New York State in the USA declared polio a public health emergency [19], the war in Ukraine intensified [20], devastating flooding was seen in Pakistan [21], and geographically-specific concerns such as Marburg disease in Ghana [22], and Ebola outbreaks in Sudan [23] and Uganda [24] have contributed to people's sense of feeling overwhelmed and fatigued with health emergencies and health information. In September 2022, the WHO Regional Director for Europe, Dr Hans Kluge, declared the European Region was in a ‘permacrisis’ due to the multiple and ongoing emergencies [25].

### Social listening and integrated analysis for infodemic preparedness and response

Social listening and integrated analysis to produce infodemic insights is the first step in managing the infodemic [26]. It can help to identify the concerns and narratives expressed by users, including information voids, misinformation, or periods of overwhelming amounts of information. During health emergencies, social media can be an important communication tool, yet it can also provide a platform for the spread of misinformation [27]. One analysis of data from 24 countries found social media was the most used source for COVID-19 information for those aged 18–40 years [28]. Narrative analysis of social media data can provide an important data source yet it is important to combine multiple data sources in a process of integrated analysis for infodemic insights [29]. This can enable the analyst to formulate actionable infodemic insights to guide emergency response and inform epidemic management and prevention strategies [29]. Throughout the pandemic the WHO COVID-19 Incident Management Support Teams globally and regionally have worked with an external service provider to develop weekly digital social listening reports to inform infodemic response to the pandemic [30]. The team was detecting regular expressions of pandemic fatigue in the publicly available narratives, both focused directly on pandemic fatigue, and those interwoven with other narratives. These narratives appeared to increase in volume and intensity as the mpox emergency started to evolve, and as other crises impacted different geographical regions on a more local level.

Defining expressions of pandemic fatigue is complex as it can manifest differently in different situations and contexts. The digital information ecosystem plays a role in how pandemic fatigue narratives are amplified, shared and received individually and through online communities and networks, and understanding this is important in analyzing the infodemic for prevention and preparedness. There is currently little known about how users express pandemic fatigue through sentiment and narratives on social media and how they can be addressed by the health authorities. To further explore and understand how these narratives were being expressed, this analysis was undertaken, seeking to identify and classify pandemic fatigue narratives into themes. This study aims to add to the overall understanding of pandemic fatigue by exploring and reporting on key narrative themes associated with expressions of pandemic fatigue by users on digital platforms.

## Methods

### Data collection and analysis

Publicly available data was gathered in English from online sources using Meltwater [31] and CrowdTangle [32]. These tools aggregated data from multiple social media platforms including Twitter, Facebook, Instagram, YouTube, TikTok, Pinterest, Product Reviews, Twitch, blogs & forums, as well as online news content that has resonated in the platforms. Post content was extracted alongside basic metadata including date, country, author and engagement. No identifiable data was included in reporting and quotations used to illustrate identified narratives were paraphrased to protect against retrievability of specific posts. The wide range of data sources, including popular social media platforms, was included to ensure breadth of coverage. Initial data sorting was two-fold. First, data were sorted by highest levels of engagement (numbers of comment replies, reactions and shares a post received), which meant that top amplified content that was most likely to resonate with social media users was sorted first. Reposts, quoted posts and forwarded posts were included in the dataset and deduplication did not occur. Prioritisation was given to items which had a high proportion of comments, shares or reposts as this indicates that people wanted content to be seen by their online following or felt strongly enough to leave a comment (rather than add a passive “like” to content). A top-level analysis was then conducted on the highest engaged subset of this dataset which was then analysed and assessed by expert global analysts to identify dominant narratives from the data, to indicate where conversations were taking place and the context around those conversations. For this analysis, data focused on the period between 23 June to 21 September 2022.

A PHEIC is a designation by the WHO implying a situation is serious, sudden, unusual or unexpected, carries implications for public health beyond the affected State’s national border and may require immediate international action [33]. Due to an apparent increase in pandemic fatigue narratives around the time of the mpox PHEIC announcement, we chose to start the search one month prior to the WHO Director General declaring mpox a PHEIC on the 23rd of July. Descriptive analysis was used to describe data distribution, including the number of identified posts each week, and the average across the time-period.

### Defining search terms

To generate a dataset that included health emergencies other than COVID-19, we broadened the aforementioned definition of pandemic fatigue to "demotivation to follow recommended protective behaviours, overwhelm with health advice, or information avoidance, in response to sustained emergencies (ie COVID-19), or multiple emergencies (ie mpox in particular, but also polio, climate change, Marburg disease etc.).” Defining the search terms for pandemic fatigue was the first step in preparing the analysis. Given the complexity of defining the way people express pandemic fatigue, this was an iterative process. Two analysts were involved in defining and testing the search. One analyst did the initial draft of keywords, data cleaning and iteration based on results. A second analyst then reviewed the keywords and results to validate breadth and relevance and final searches were determined via discussion. Users do not talk about pandemic fatigue by mentioning the phase “pandemic fatigue” so a range of search terms were trialled to identify expression, and the results reviewed for relevance after each trial. Initially the analysts trialled phrases assumed to be associated with pandemic fatigue, such as “I’m sick of”, “tired of hearing”, “over this”, “people have had enough”, “want to live again” among many others. These terms tended to bring up instances of users dismissing pandemic fatigue, rather than expressing it. This was a useful insight for the research team, and it added to the experience from the routine weekly infodemic insights analysis that pandemic fatigue was a widespread and growing sentiment. The final list was informed by work done throughout the COVID-19 pandemic producing digital insights reports and knowledge of how conversations, and associated keywords, have evolved. A total of 483 root terms and phrases were included and rounds of testing, iteration and validation were conducted (see Additional file 1 for full list).

### Thematic analysis of narratives

Thematic analysis describes a process of seeking understanding of data through organisation and coding data into themes [34]. There are six steps to thematic analysis of qualitative data: data familiarisation, initial generation of codes, searching and identifying themes, reviewing themes, defining and naming the theme, final analysis and report production [35].

For this project, the lead researcher conducted an inductive thematic analysis on the dominant narratives identified through the initial search as being about pandemic fatigue following the above steps. This involved initially reading and rereading narratives and posts to identify potential themes. Data were coded manually, and themes refined and reviewed throughout the analysis process. Defining and naming the themes was then completed alongside a thorough review of content coded to each theme. To ensure confirmability, and reduce any researcher bias in coding, this was then critically reviewed by a second researcher with the final set of themes and data coding confirmed via discussion between the two researchers.

## Results

### Data collection

**Fig. 1:**
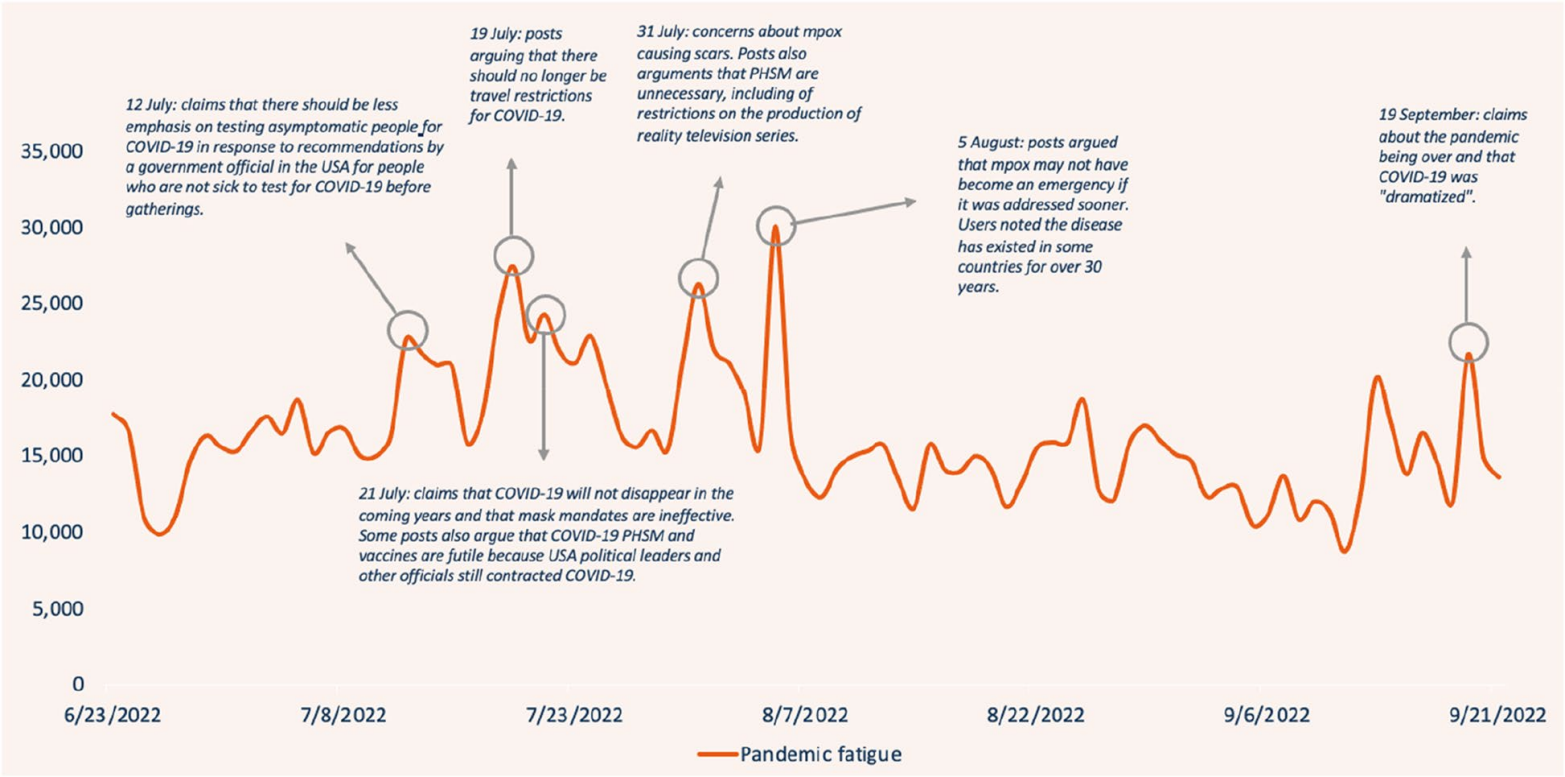
Pandemic fatigue related posts with top-level analysis of narratives driving the peaks in data volume

There were 1,484,042 social media posts identified during the time-period that included the defined search terms. A qualitative process of narrative identification was undertaken prioritising the topmost engaged with data. This involved looking for linkages between posts, reoccurring trends, and amplification of content. Due to the nature of this iterative process, only 1% of individual posts were included in this analysis as it included a top-level overview using a sub-set of the data with the highest engagement. Due to the rounds of testing and iteration, only a small amount of irrelevant content was identified. Figure 1 shows the volume of posts over time, with the main narratives driving volume peaks identified.

Table 1 shows the number of identified social media posts per week throughout the time-period. The average number of social media posts per week was 114,147 (std 20,000). The bolded rows (week 3–6) depict the 4 weeks around the time of the mpox PHEIC announcement on the 23rd of July 2022, which shows a consistent higher than average numbers of identified posts for this time.

**Table 1:** Average number of weekly identified social media posts

	**Week starting**	**No of posts**
1	23/6/2022	97,209
2	30/6/2022	115,513
3	**7/7/2022**	**124,017**
4	**14/7/2022**	**149,861**
5	**21/7/2022**	**141,871**
6	**28/7/2022**	**141,840**
7	4/8/2022	116,986
8	11/8/2022	99,898
9	18/8/2022	101,434
10	25/8/2022	107,521
11	1/9/2022	88,210
12	8/9/2022	92,601
13	15/9/2022	107,081

### Thematic analysis

Users talked about and expressed pandemic fatigue in a range of different ways. Six main themes emerged from the narrative analysis relating to expression of pandemic fatigue, and one theme of counter narratives against pandemic fatigue. These themes are described below.

1. Sustained emergency driving narratives preventative measures are ineffective
2. Anger, apathy or disinterest towards preventative measures
3. Information avoidance or dismissal
4. Overwhelmed with multiple or sustained emergencies
5. Comparisons with multiple or sustained emergencies
6. Distrust in health and other authorities
7. Counter narratives against pandemic fatigue

#### Sustained emergency driving narratives preventative measures are ineffective

The prolonged and evolving nature of the COVID-19 pandemic drove narratives in this category. Narratives focused on the persistence of COVID-19, despite all the measures put in place, as evidence that these preventative measures, including vaccination, were ineffective. This included PHSM such as mask-wearing and lockdowns being seen as ineffective and of little use, and there was a particularly strong opposition identified to children wearing masks. This was due to claims the risk of COVID-19 to children had been overstated and that there was no evidence regarding masks. Users made statements such as *“Shouldn’t it be clear that vaccines & masks DON’T WORK”*, *“If the jabs were effective, why are covid infections rising???”,* and *“Lockdowns all the time were a waste of our time”.*

#### Anger, apathy or disinterest towards preventative measures

Different to the previous theme, narratives in this theme expressed indifference, apathy, disinterest or anger towards preventative measures. Observed in this theme were narratives regarding PHSM compliance (*“hardly anyone cares and is following the rules”),* calls not to normalise PHSM and vaccine passports *(“[Don’t want] faceless societies”),* and general demotivation for measures, with users asking,* “Why are we still wearing masks?”, “Why are we still testing for COVID?”.*

#### Information avoidance or dismissal

This theme was characterised by users dismissing information, often by a ‘Yawn’ or an eye-roll emoji  or exhibiting information avoidance. Reactions to reports of new COVID-19 variants being considered a threat were likely to prompt responses expressing sarcasm, *“Here we go again! Yawn* *”* or a dismissive lack of concern “Breaking news: there’s a new “scary” covid variant. *sigh*”. Users asked how long the media would keep reporting COVID-19 information, making statements such as *“So over it, Nobody cares”*. Similarly, reports about mpox being declared a public health emergency were met with *“Yawn”.*

#### Overwhelmed with multiple or sustained emergencies

The convergence of multiple emergencies, or the prolonged nature of the COVID-19 pandemic led users to express feelings of being overwhelmed. This included feelings of helplessness and frustration at the continued impact on daily life. Users referred to being tired of pretending things were ok, or of having to suppress all their concerns and fears to enable them to continue with daily life, “This place is a s**tshow right now… I’m exhausted from pretending [it’s okay]”. Also, in this theme were narratives identified as questioning ongoing emergency measures as the COVID-19 pandemic and other emergencies continue to impact on user’s daily lives.

#### Comparisons with multiple or sustained emergencies

Comparisons between health emergencies were frequently used to downplay the level of risk of a particular emergency. This included challenging the emergency status, challenging the use of PHSM, or linking to conspiracy theories. The impact of COVID-19 was unfavourably compared to other emergencies with users stating that global issues such as starvation, seasonal influenza, heart disease, injury and cancer contributed more to the burden of mortality and morbidity than COVID-19. As an example, one user stated, “I’m tired of reading about Covid and now this monkeypox cr*p. More people died of hunger than from COVID-19”. Another compared COVID-19 deaths to heart disease stating, *“Almost 2,000 per day die from heart disease, what on earth are we doing?”* Re-occurring questioning of the climate change emergency was observed, including that climate change was a scam just like COVID-19, that climate change would be used to keep citizens living in fear as COVID-19 fatigue sets in, and that climate change will result in the next round of lockdowns.

#### Distrust in health and other authorities

Narratives in this theme expressed distrust towards health or other authorities. These were predominantly observed around the perceived handling of the COVID-19 pandemic or other health emergencies. Examples included users stating, “*The government and media lied”*, and *“Government doesn’t want to give up its new powers”*. Also observed in this theme were multiple conspiracy-related narratives about PHSM recommendations being social control by authorities, the media being used to drive fear, and the mpox outbreak being the next step in the ‘*plandemic’*. Other narratives referred to authorities’ scaremongering over new variants. Interestingly, in this theme were expressions of distrust driven by disappointment of perceived PHSM or vaccine efficacy. Promises made early in the pandemic about how the vaccine would prevent transmission or would prevent illness, were used to express distrust in officials claims about subsequent boosters and to question the science.

#### Counter narratives against pandemic fatigue

Counter narratives came from users who were expressing disappointment at the complacencies of others, or other people’s expression of pandemic fatigue and associated reported behaviours. This was largely in relation to users feeling judged for continuing with PHSM, or also being tired of continuing with PHSM, but needing to continue nonetheless. Users who identified as being high-risk for COVID-19, or immunocompromised were observed in this theme. Some examples included users stating, “*We are SO bored of covid too*”, or “*We are over Covid too, but it’s here, and to keep our families safe we need to face it*.” Some users reported being dismissed by family members or experiencing acts of bullying and mockery at their continued adherence to PHSM, with one user asking, *“Why are people bullying those people who wear masks?”.*

## Discussion

This paper has identified different ways users express pandemic fatigue on social media. Although pandemic fatigue is a significant concern for health authorities, this paper is the first that we are aware that has sought to define and categorise expressions of pandemic fatigue by digital online users in relation to health emergencies. The themes we identified provide insight into the different ways fatigue is expressed and how it is often conflated with other narrative types. This paper adds several interesting findings to the knowledge of pandemic fatigue.

While pandemic fatigue narrative volume increased around the time of the mpox PHEIC announcement, conversation was not always directly related to mpox, as some of the peaks were driven by COVID-19 content, and this could have been related to other factors. However, this narrative crossover was also observed in the themes of ‘Overwhelmed with multiple or sustained emergencies’ and ‘Comparisons with multiple or sustained emergencies’ where different health emergencies were driving fatigue across adjacent issues. There were instances of users bringing several health emergencies together to express feeling overwhelmed by the sheer number of crises the world is facing. Other research has reported on ways of building community resilience to recover from the COVID-19 pandemic, including through social media [36], and this is an important area of research. There is a need for a nuanced approach to information delivery during the time of emerging health concerns. A range of factors impact how users receive and response to information such as psychological factors, perceived credibility of source, and user digital and health literacy [37]. Further, the social and commercial determinants of health influence how people experience and interact with the information environment [38]. Rather than just adding more information, researchers and health authorities can consider how information is likely to be received and be guided by principles of codesign and community engagement.

Infodemic insights can be used to understand community responses and reactions. As well as offering immediate insights for actions during an emergency, analyzing patterns of infodemic insights and narratives overtime, such as this research, can offer insights into user expression and help guide prevention, preparedness and readiness. Expressions of pandemic fatigue were interwoven with distrust in authorities, demotivation to use PHSM and feelings of being overwhelmed, angry or dismissive. These are important considerations when a health emergency is evolving, and targeted work is needed to respond effectively. Research has shown that greater trust in government is associated with greater acceptance of health guidelines [39]. Analysis of trust-related narratives on digital platforms during an emerging health emergency found that these narratives manifested in different ways and that there were more mistrust related narratives associated with a country with a lower trust in government score [40]. Trust in authorities during a health emergency is complex and multi-faceted [41] and building trust and health literacy prior to an emergency are key preventative strategies. Other research has found an association with high levels of social media exposure and information overload, information anxiety, and information avoidance [42]. Although we did not look at volume of narratives by theme for this study, looking at patterns of narrative expressions by theme (i.e., by information avoidance or dismissal) mapped to total volume and key events may provide further interesting insights.

The use of emoji, in particular eyeroll, yawn and confused faces, to express pandemic fatigue shows the challenges in finding and identifying narratives by using only text-based analysis. Further research may be enhanced by including emoji, meme, image and video analysis. The complexity in defining the search strategy for this research shows the nuanced approach that is needed, and our search strategy benefited from rounds of iteration and testing. The work done in defining both pandemic fatigue search terms as well as themes of expression will be useful for further research. More research is needed in languages other than English and over a longer timeframe.

Guidance for action on mitigating pandemic fatigue includes risk communication and community engagement and consultation, risk reduction, and acknowledgement of the situations [6]. As the science and public health recommendations change throughout a health emergency, there is a need to carefully consider how the information environment impacts users, to effectively manage narratives regarding trust in health authorities. There are lessons to be learnt from the COVID-19 pandemic that can be applied to future infodemic planning. Infodemic management is a key component in the recently released WHO report, ‘Strengthening the global architecture for health emergency prevention, preparedness, response and resilience’ [43]. Using digital and offline data to create infodemic insights for action, guided by appropriate frameworks, can help to enhance timely response and work towards the prevention and mitigation of the infodemic.

### Limitations

The themes we identified in our analysis describe the different way users’ express pandemic fatigue, however, our work was only conducted over a short time-period and only from online sources that had publicly accessible data. As this was an exploratory exercise, with significant testing of keywords, the search was conducted only in the English language. The analysis was done on the dataset as a whole and we did not breakdown data or themes by geographical origin or social media platform. There is a need for broader investigations including in other languages and with offline sources. This research identified six themes of pandemic fatigue expression, but additional, or different themes may be identified using longer search periods or refined search terms. Due to the various ways user’s express narratives of fatigue, and how they cross-over with other more specific narratives, defining and understanding pandemic fatigue is complex. This work adds to the evidence by outlining some themes of expression.

## Conclusion

This paper is the first that we are aware that has sought to define and categorise expression of pandemic fatigue by digital online users during a defined period. It has shown the different ways pandemic fatigue is expressed and how it is interwoven with issues of trust, PHSM acceptance and users expressing being overwhelmed. There are clear pathways for future research in this area, in particular looking to understand how this concept is expressed in other languages, in offline settings and what impact pandemic fatigue has on actual PHSM, vaccines, treatments, or diagnostics uptake and adherence. As we move to strengthen the global architecture for health emergency preparedness, prevention, readiness, and resilience, better understanding the implications of the information environment on user’s perceptions, questions and concerns is vital.

### Supplementary Information

**Additional file 1: Appendix 1.** Keywords and phrases for search strategy.

## Data Availability

The datasets generated and/or analysed during the current study are available from the corresponding author on request.

## Abbreviations

PHSM: Public Health and Social Measures
WHO: World Health Organization
PHEIC: Public Health Emergency of International Concern

## References

[1] World Health Organization. 2020. WHO Director-General's opening remarks at the media briefing on COVID-19 - 11 March 2020. https://www.who.int/director-general/speeches/detail/who-director-general-s-opening-remarks-at-the-media-briefing-on-covid-19---11-march-2020.

[2] Briand S, Hess S, Nguyen T, Purnat TD, Purnat TD, Nguyen T, Briand S (2023). Infodemic Management in the Twenty-First Century. Managing infodemics in the 21st century : addressing new public health challenges in the information ecosystem.

[3] Purnat TD, Vacca P, Czerniak C, Ball S, Burzo S, Zecchin T, Wright A, Bezbaruah S, Tanggol F, Dubé È, Labbé F, Dionne M, Lamichhane J, Mahajan A, Briand S, Nguyen T (2021). Infodemic signal detection during the COVID-19 pandemic: development of a methodology for identifying potential information voids in online conversations. JMIR Infodemiol.

[4] Chaney SC, Benjamin P, Mechael P. Finding the signal through the noise. GAVI, UNICEF, WHO, VDH, Health Enabled 2021. https://www.gavi.org/sites/default/files/2021-06/Finding-the-Signal-Through-the-Noise.pdf.

[5] World Health Organization. 10 proposals to build a safer world together – strengthening the global architecture for health emergency preparedness, response and resilience: draft for consultation. Geneva: WHO; 2022: CC BY-NC-SA 3.0 IGO. https://cdn.who.int/media/docs/default-source/emergency-preparedness/who_hepr_june30draftforconsult.pdf?sfvrsn=e6117d2c_4&download=true.

[6] WHO Regional Office for Europe. Pandemic fatigue. Reinvigorating the public to prevent COVID-19. Copenhagen: WHO: 2020. https://apps.who.int/iris/bitstream/handle/10665/335820/WHO-EURO-2020-1160-40906-55390-eng.pdf.

[7] Reicher S, Drury J (2021). Pandemic fatigue? How adherence to covid-19 regulations has been misrepresented and why it matters. BMJ.

[8] Zarowsky Z, Rashid T (2023). Resilience and wellbeing strategies for pandemic fatigue in times of Covid-19. Int J Appl Posit Psychol.

[9] MacIntyre CR, Nguyen P-Y, Chughtai AA, Trent M, Gerber B, Steinhofel K, Seale H (2021). Mask use, risk-mitigation behaviours and pandemic fatigue during the COVID-19 pandemic in five cities in Australia, the UK and USA: a cross-sectional survey. Int J Infect Dis.

[10] Labrague LJ (2021). Pandemic fatigue and clinical nurses’ mental health, sleep quality and job contentment during the covid-19 pandemic: the mediating role of resilience. J Nurs Manag.

[11] Labrague LJ, Ballad CA (2021). Lockdown fatigue among college students during the COVID-19 pandemic: predictive role of personal resilience, coping behaviors, and health. Perspect Psychiatr Care.

[12] Bodas M, Kaim A, Velan B, Ziv A, Jaffe E, Adini B (2022). Overcoming the effect of pandemic fatigue on vaccine hesitancy-will belief in science triumph?. J Nurs Scholarsh.

[13] Monmouth University Poll. 2021. National: Most Americans ‘Worn out’ by COVID. [Press Release] 15 December 2021. Available at: https://www.monmouth.edu/polling-institute/documents/monmouthpoll_us_121521.pdf/.

[14] Johns Hopkins University CSSE COVID-19 data. Daily new confirmed COVID-19 cases per million people. In: University JH, editor. Our world in data2022.

[15] Lockyer B, Islam S, Rahman A, Dickerson J, Pickett K, Sheldon T, Wright J, McEachan R, Sheard L (2021). Understanding COVID-19 misinformation and vaccine hesitancy in context: findings from a qualitative study involving citizens in Bradford. UK Health Expect.

[16] UNICEF. 2022. COVID-19 pandemic fuels largest continued backslide in vaccinations in three decades. [Press Release] 14 July 2022. Available at: https://www.unicef.org/press-releases/WUENIC2022release.

[17] Glenza J. Slow response to monkeypox exposes ‘tired, overworked’ US health agencies. 2022 The Guardian. 2022. https://www.theguardian.com/world/2022/aug/01/monkeypox-us-virus-vaccine-health-response

[18] World Health Organization. 2022. WHO Director-General declares the ongoing monkeypox outbreak a Public Health Emergency of International Concern. [Press Release] Available at: https://www.who.int/europe/news/item/23-07-2022-who-director-general-declares-the-ongoing-monkeypox-outbreak-a-public-health-event-of-international-concern.

[19] Tanne JH (2022). Polio emergency declared in New York state over virus found in wastewater. BMJ.

[20] The Guardian. Russia-Ukraine war at a glance: A summary of the most significant developments in the Ukraine-Russia conflict. 2022. The Guardian. 2022. https://www.theguardian.com/world/series/russia-ukraine-war-at-a-glance

[21] Trenberth K. 2022’s supercharged summer of climate extremes: How global warming and La Niña fueled disasters on top of disasters. 2022 The Conversation. 2022. https://theconversation.com/2022s-supercharged-summer-of-climate-extremes-how-global-warming-and-la-nina-fueled-disasters-on-top-of-disasters-190546

[22] Araf Y, Maliha ST, Zhai J, Zheng C. Marburg virus outbreak in 2022: a public health concern. Lancet Microbe10.1016/S2666-5247(22)00258-0 10.1016/S2666-5247(22)00258-0 36209757

[23] World Health Organization. Ebola: Mbandaka, Equateur Province, Democratic Republic of the Congo, 2022. 2022 WHO. 2022. https://www.who.int/emergencies/situations/ebola-équateur-province-democratic-republic-of-the-congo-2022

[24] World Health Organization. Ebola Disease caused by Sudan virus - Uganda. 2022 WHO. 2022. https://www.who.int/emergencies/disease-outbreak-news/item/2022-DON410

[25] WHO Regional Office for Europe. Statement: The European Region is in a “permacrisis” that stretches well beyond the pandemic, climate change and war. 2022 WHO Regional Office for Europe. 2022. https://www.who.int/europe/news/item/27-09-2022-statement-the-european-region-is-in-a-permacrisis-that-stretches-well-beyond-the-pandemic-climate-change-and-war

[26] Rubinelli S, Purnat TD, Wilhelm E, Traicoff D, Namageyo-Funa A, Thomson A, Wardle C, Lamichhane J, Briand S, Nguyen T (2022). WHO competency framework for health authorities and institutions to manage infodemics: its development and features. Human Res Health..

[27] Cuello-Garcia C, Pérez-Gaxiola G, van Amelsvoort L (2020). Social media can have an impact on how we manage and investigate the COVID-19 pandemic. J Clin Epidemiol.

[28] Blandi L, Sabbatucci M, Dallagiacoma G, Alberti F, Bertuccio P, Odone A. Digital information approach through social media among Gen Z and millennials: the global scenario during the covid-19 pandemic. Vaccines 2022;2022. 10.3390/vaccines10111822 PMC9696549 36366331

[29] World Health Organization, United Nations Childrens Fund. How to Build an Infodemic Insights Report in 6 Steps Geneva: WHO & UNICEF 2023: Licence: CC BY-NC-SA 3.0 IGO. https://www.who.int/publications/i/item/9789240075658.

[30] Purnat TD, Nguyen T, Ishizumi A, Yau B, White B, Cecchini S, Samuel R, Hess S, Bezbaruaha S, Briand S (2022). Delivering actionable infodemic insights and recommendations for the COVID-19 pandemic response. WHO Wkly Epidemiol Record.

[31] Meltwater. n.d. Meltwater. https://explore.meltwater.com/brand-media-intelligence.

[32] CrowdTangle Team. 2021. CrowdTangle. Facebook, Menlo Park, California, United States. https://www.crowdtangle.com.

[33] WHO. 2019. Emergencies: International health regulations and emergency committees. https://www.who.int/news-room/questions-and-answers/item/emergencies-international-health-regulations-and-emergency-committees.

[34] Braun V, Clarke V (2006). Using thematic analysis in psychology. Qual Res Psychol.

[35] Bryman A. Social research methods 2nd ed. Oxford: Oxford University Press; 2004. 0199588058: 0199588058

[36] Xie L, Pinto J, Zhong B (2022). Building community resilience on social media to help recover from the COVID-19 pandemic. Comput Human Behav.

[37] Sylvia Chou WY, Gaysynsky A, Cappella JN (2020). Where we go from here: health misinformation on social media. Am J Public Health.

[38] White B, Phuong L, Roach J, Teggelove N, Wallace H. Pandemics, infodemics and health promotion. Health Promot J Austr. 2022;n/a(n/a). 10.1002/hpja.644. 10.1002/hpja.644 PMC9353363 35906964

[39] Bollyky TJ, Hulland EN, Barber RM, Collins JK, Kiernan S, Moses M, Pigott DM, Reiner RC, Sorensen RJD, Abbafati C, Adolph C, Allorant A, Amlag JO, Aravkin AY, Bang-Jensen B, Carter A, Castellano R, Castro E, Chakrabarti S, Combs E, Dai X, Dangel WJ, Dapper C, Deen A, Duncan BB, Earl L, Erickson M, Ewald SB, Fedosseeva T, Ferrari AJ, Flaxman AD, Fullman N, Gakidou E, Galal B, Gallagher J, Giles JR, Guo G, He J, Helak M, Huntley BM, Idrisov B, Johanns C, Le Grand KE, Letourneau ID, Lindstrom A, Linebarger E, Lotufo PA, Lozano R, Magistro B, Malta DC, Månsson J, Mantilla Herrera AM, Marinho F, Mirkuzie AH, Mokdad AH, Monasta L, Naik P, Nomura S, O'Halloran JK, Odell CM, Olana LT, Ostroff SM, Pasovic M, Passos VMDA, Penberthy L, Reinke G, Santomauro DF, Schmidt MI, Sholokhov A, Spurlock E, Troeger CE, Varavikova E, Vo AT, Vos T, Walcott R, Walker A, Wigley SD, Wiysonge CS, Worku NA, Wu Y, Wulf Hanson S, Zheng P, Hay SI, Murray CJL, Dieleman JL (2022). Pandemic preparedness and COVID-19: an exploratory analysis of infection and fatality rates, and contextual factors associated with preparedness in 177 countries, from Jan 1, 2020, to Sept 30, 2021. Lancet..

[40] White BK, Lavery L, Ishizumi A, Wright A, Foley T, Nguyen T, Briand S, Machiri S, Hassan N, Pastorino A, Purnat TD (2023). Infodemic insights on Trust in a Health Emergency: a narrative deep-dive. Stud Health Technol Inform.

[41] Sopory P, Novak JM, Day AM, Eckert S, Wilkins L, Padgett DR, Noyes JP, Allen T, Alexander N, Vanderford ML, Gamhewage GM (2022). Trust and public health emergency events: a mixed-methods systematic review. Disas Med Public Health Prep.

[42] Soroya SH, Farooq A, Mahmood K, Isoaho J, Zara S-E (2021). From information seeking to information avoidance: Understanding the health information behavior during a global health crisis. Inform Processing Manage..

[43] World Health Organization. Strengthening the global architecture for health emergency prevention, preparedness, response and resilience. Geneva: WHO 2023: Licence: CC BY-NC-SA 3.0 IGO. https://cdn.who.int/media/docs/default-source/emergency-preparedness/who_hepr_wha2023-21051248b.pdf?sfvrsn=a82abdf4_3&download=true.

[44] franzke as, Bechmann A, Zimmer M, Ess C, Association of Internet Researchers. Internet Research: Ethical Guidelines 3.0. 2020. https://aoir.org/reports/ethics3.pdf.

