# Supplementary material for: Expressions of pandemic fatigue on digital platforms: a thematic analysis of sentiment and narratives for infodemic insights
Source: BMC Public Health. 2024 Mar 5;24:705. doi: 10.1186/s12889-024-17718-4 (PMC10916327; doi:10.1186/s12889-024-17718-4)

**Appendix 1. Keywords and phrases for search strategy**

* Indicates search for variants of a word with a particular stem facilitated


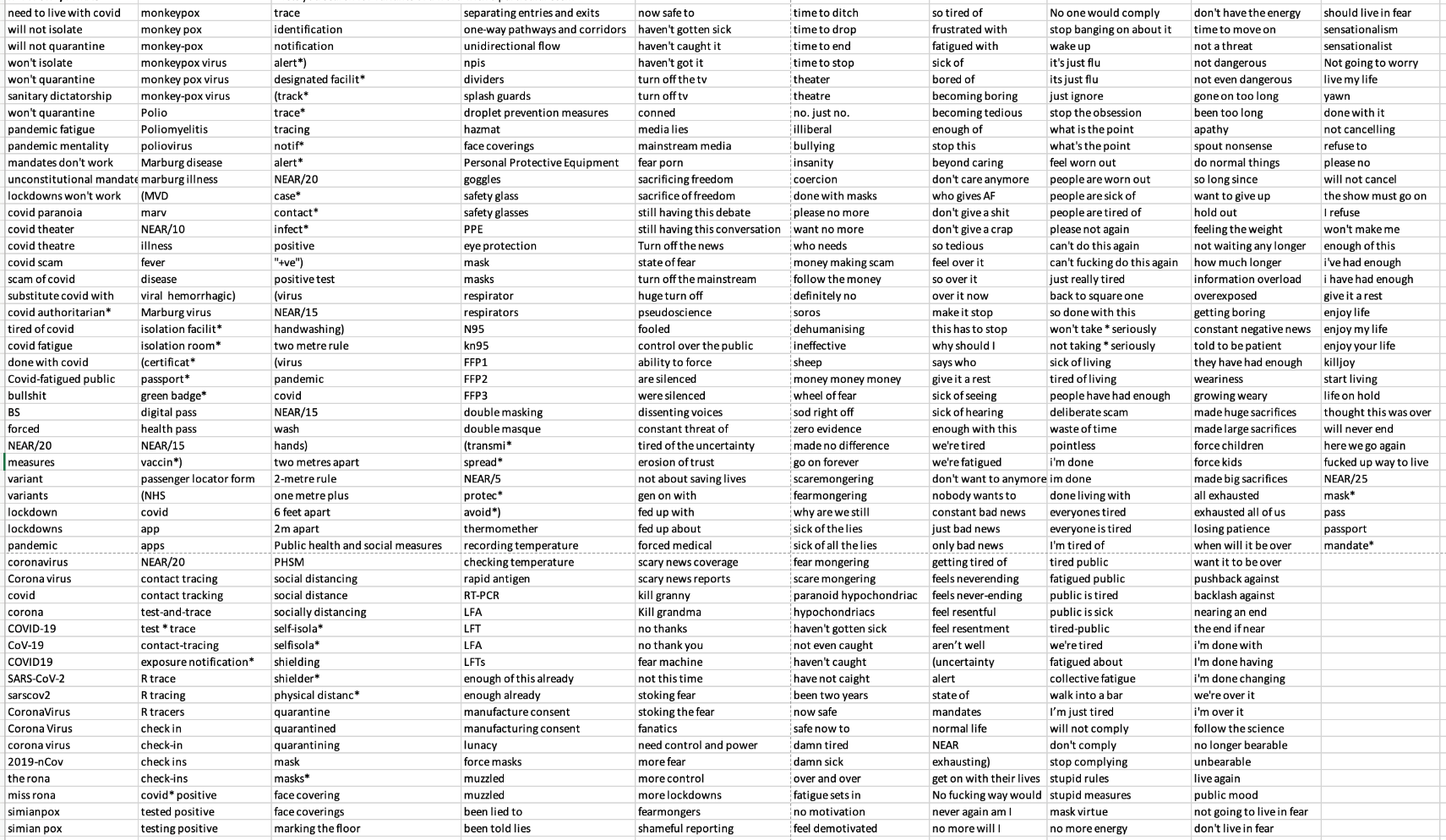

Supplement: Supplementary file 1 — Additional file 1: Appendix 1. Keywords and phrases for search strategy. [file 12889_2024_17718_MOESM1_ESM.docx]
